# Monitoring of Damage in Composite Structures Using an Optimized Sensor Network: A Data-Driven Experimental Approach

**DOI:** 10.3390/s23042290

**Published:** 2023-02-18

**Authors:** Sandris Ručevskis, Tomasz Rogala, Andrzej Katunin

**Affiliations:** 1 Institute of Materials and Structures, Riga Technical University, Kipsalas Iela 6A, LV-1048 Riga, Latvia; 2 Department of Fundamentals of Machinery Design, Faculty of Mechanical Engineering, Silesian University of Technology, Konarskiego 18A, 44-100 Gliwice, Poland

**Keywords:** structural health monitoring, delamination detection, optimal sensor placement, modal analysis, composite structure

## Abstract

Due to the complexity of the fracture mechanisms in composites, monitoring damage using a vibration-based structural response remains a challenging task. This is also complex when considering the physical implementation of a health monitoring system with its numerous uncertainties and constraints, including the presence of measurement noise, changes in boundary and environmental conditions of a tested object, etc. Finally, to balance such a system in terms of efficiency and cost, the sensor network needs to be optimized. The main aim of this study is to develop a cost- and performance-effective data-driven approach to monitor damage in composite structures and validate this approach through tests performed on a physically implemented structural health monitoring (SHM) system. In this study, we combined the mentioned research problems to develop and implement an SHM system to monitor delamination in composite plates using data combined from finite element models and laboratory experiments to ensure robustness to measurement noise with a simultaneous lack of necessity to perform multiple physical experiments. The developed approach allows the implementation of a cost-effective SHM system with validated predictive performance.

## 1. Introduction

Polymer matrix composite (PMC) materials, in addition to their superior mechanical properties and resistance to numerous environmental conditions and chemical solutions, are prone to various types of structural damage that appear during the operation of structures and components made of them. The most typical types of operational damage of such materials are matrix cracks, delamination and impact damage, among others. Due to their specific composition, PMCs are especially susceptible to different types of damage. In numerous structures, such as aircraft, marine and civil structures, a timely detection of damage is crucial for structural integrity and safety; therefore, continuous control of a structural condition is often essential for effective operation of PMC structures in such applications.

Due to the significant increase in the application of composite materials in numerous industries, primarily for load bearing structures, structural health monitoring (SHM) systems have gained much attention both from the viewpoints of scientific developments and their practical application [1]. Such systems are usually based on strain sensors, piezoelectric elements or optical fibers with fiber Bragg gratings (FBG). Numerous implementations of such systems used to monitor damage in PMCs can be found for structural applications in aircraft, marine and civil structural applications. The authors of [2] developed an SHM system for aircraft wing inspection, Baker et al. [3] developed a strain-based SHM system to monitor the repaired F-111C aircraft wing, and the authors of [4] presented several examples of SHM systems for various structural elements and components. In [5], an SHM system for aircraft structures using piezoelectric transducers was presented. An overview of such systems using piezoelectric transducers can be found in [6]. Some examples of the implementation of FBG-based SHM systems for monitoring boat hulls can be found in studies by Mieloszyk et al. [7] and Min et al. [8]. The SHM systems developed for civil engineering applications are summarized in review papers [9,10,11,12], while some previous developments of such systems for monitoring crack propagation in reinforced concrete bridges are presented in [13,14]. As has been mentioned in numerous studies, the high cost of a sensor network, especially for large structures, remains the primary aspect which limits the wide applicability of SHM systems. Therefore, one of the emerging problems that deserves attention is the optimization of the number and location of SHM systems, whose main aim is to effectively monitor structural damage with a possibly small number of sensors.

As discussed in our previous study [15], the oldest and most widespread SHM approach is based on monitoring the vibration response of a structure, which is considered in many applications, including composite beams [16] and plates [17,18,19]. An overview of the variety of approaches to the problem of optimal sensor placement (OSP) in vibration-based SHM presented in [15] clearly shows the necessity of developing effective algorithms focused on minimizing the number of sensors in the measurement network without losing the quality and accuracy of structural damage. The fundamentality of solving an OSP problem in SHM systems is underlined in many recent studies; see, e.g., [20,21,22]. Taking into account the variety of factors that influence OSP, which include numerous uncertainties, assumed criteria, optimization algorithms and strategies, the problem is not trivial, especially when it is implemented physically as an SHM system on a composite structure. In this case, additional uncertainties, such as variation of local stiffness and material properties due to possible manufacturing defects, noise in acquired signals, sensor accuracy, physical constraints on sensor placement, etc., need to be taken into consideration.

In our previous study [15], we focused on the appropriate selection of sensor network. The networks were a result of the application of different algorithms of optimal sensor networks, which were evaluated using some of the criteria. The algorithms which provided sensor networks based on the access to healthy condition strain maps only of the analyzed composite plate. Apart from the main criteria of optimal placement, the minimum sufficient number of sensors and used modes were also factors that were taken into account. The effectiveness of this approach was evaluated on the basis of well-trained classifiers which were able to evaluate the response of sensor networks to damage detection and to effectively detect delamination with an arbitrary location.

This paper is a continuation of a previous computational study, which is focused on the practical implementation of the developed OSP procedure in PMC plates with the analysis of the effectiveness of this procedure in the presence of numerous uncertainties, constraints and limitations present in physical SHM systems. These include the presence of measurement noise, changes in boundary and environmental conditions of a tested object, numerous aspects related to the manufacturing process of composite structures and the resulting homogeneity of material properties, which may affect the correctness of the damage detection procedure, and many others. In contrast to previous research studies presented in [15], which were focused on the selection of the most effective OSP procedure and based on numerical simulations only, in this study, we focus on the experimental implementation of the selected procedure. Systematic studies on the detection of delamination in PMCs using the optimized sensor network demonstrated satisfactory results. The novel data-driven experimental approach proposed in this study, which was an improvement to previous computational results [15], is robust to measurement noise and reveals a high effectiveness in damage detection due to the applied combination of both numerical and experimental results in the learning algorithm. The developed data-driven approach falls into the current trends in the development of SHM systems, a concept which was recently used, e.g., by the authors of [23]. It should be mentioned that the data used in this study were acquired from physical experiments and were additionally experimentally validated using the NDT ultrasonic testing technique. The machine learning algorithm k-NN within two supervised learning schemes is used to create classification models by learning from simulated response data. The predictive performance of the developed classification models is evaluated by introducing trained models to unseen experimental data and identifying the damage in the composite plates. The main advantage of this approach is the development of an economically reliable SHM system robust to measurement noise and changes in boundary conditions of a tested structure with an optimized number and location of sensors, which is achievable without the need to perform a limited number of physical experiments.

## 2. Materials and Methods

The following study focused on the determination of the detectability of artificially introduced delamination in a composite plate. Details on the manufacturing of the tested plates and its initial examination are presented in this section.

### 2.1. Specimen Preparation

**Figure 1:**
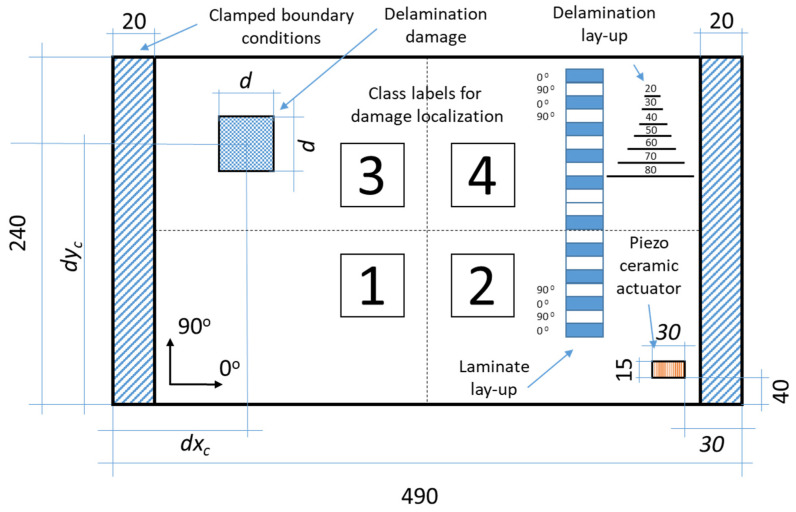
The schematic representation of the CFRP plate with an artificial delamination damage and piezo ceramic actuator.

In the study, two CFRP plates (healthy and with artificial delamination) with spatial dimensions of 490 × 240 mm were considered for experimental testing and the validation of the developed concepts and methods. The staking sequence and other dimensions are presented in Figure 1.

The plates were manufactured using the hand layup method by assembling individual prepreg layers cut from a roll of the UNIPREG^®^ Carbon non-crimp fabric of 100 g/m^2^ (Unicarbon, Kaunas, Lithuania) into a laminate structure according to the (0/90)_5s_ laminate lay-up. Artificial damage in the form of delamination simulating low-velocity impact is introduced in one of the plates by placing Teflon^®^ inserts of different sizes between the respective plies according to the scheme presented in Figure 1. The center point coordinates for all delamination inserts are as follows: *d_xc_* = 160 mm and *d_yc_* = 80 mm. The planar size of the delamination varies according to Figure 1, for instance, the size of the insert between layers 2 and 3 is *d* = 20 mm, while *d* = 80 mm for the insert between layers 8 and 9. The assembled laminates are covered with a vacuum bag and sealant tape to create mechanical pressure and cured in an oven.

### 2.2. Initial Validation Using Ultrasonic Testing

**Figure 2:**
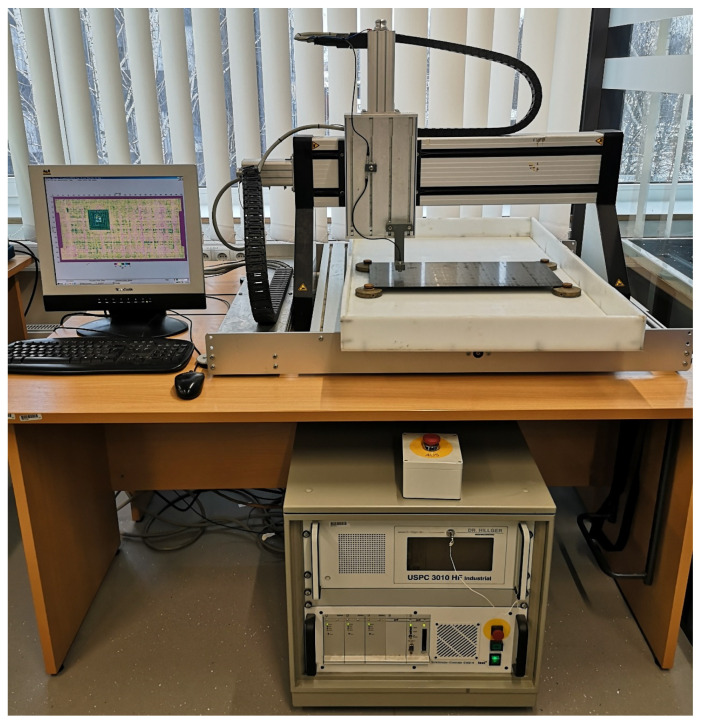
The ultrasonic experimental setup.

The quality of the manufactured plates and the introduced delamination damage was assessed using an ultrasonic non-destructive technique. Initial validation tests were performed using the USPC 3010 Industrial ultrasonic defect detector (Ingenieurbüro Dr. Hillger, Braunschweig, Germany). The scanner performed pulse-echo C-scans using a 10 MHz probe (broadband dual transmitting-receiving transducer with the focal length of 25.4 mm). The specimens were immersed in a water tank during testing and the scanning procedure was performed with a motor-controlled stepper XYZ manipulator that drives the probe (shown in Figure 2).

**Figure 3:**
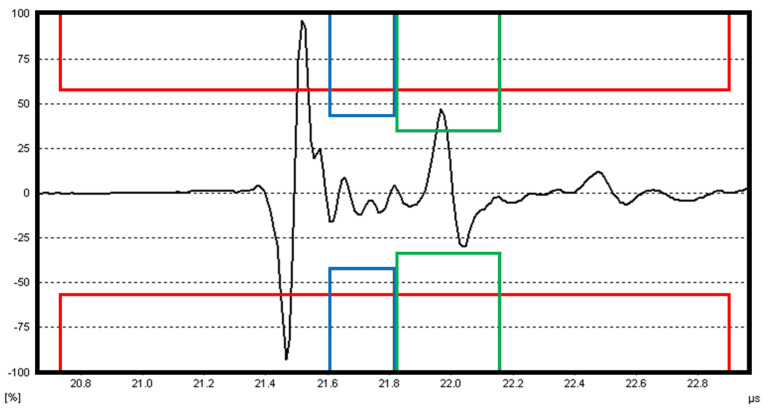
The exemplary A-scan recorded from a defect-free region of the plate.

The exemplary A-scan acquired from the intact region of the tested plate is presented in Figure 3. The dedicated Hilgus software used for evaluation of scanning results makes it possible to define 3 different gates of acquiring signals. The first gate (marked with the red box in Figure 3) captures a signal reflected from the first limiting surface. When a defect in the material is present, a part of the introduced ultrasonic wave reflects from this defect and returns to the receiving transducer. Due to this, the signal is recorded as an echo of this defect, which is marked by the blue box in Figure 3. The other part of this wave propagates through the defect to the surface of a specimen opposite to the scanned surface and reflects from it. This reflection returns to the receiver with a certain delay in the form of a backwall echo (marked with the green box in Figure 3). The depth of a defect from the scanned surface is determined based on the time-of-flight principle, i.e., based on the time delay of an ultrasonic wave represented by a distance between signals in red and blue boxes.

**Figure 4:**
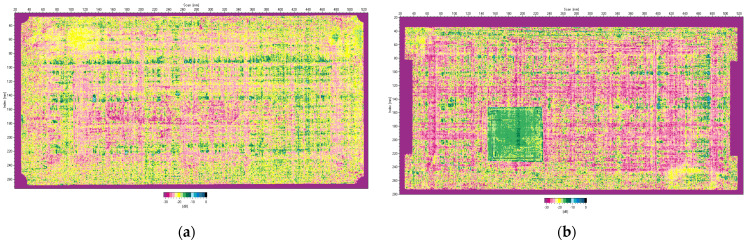
Ultrasonic C-scan (time-of-flight mode) images of the plates: (**a**) healthy plate; (**b**) plate with an artificial delamination.

The Hilgus software provides imaging of test results in A-, B-, C-, and D-scans. A typical C-scan image combines A-scan data and is plotted on a plan view of the plate. The C-scan image for the tested plates was recorded with a resolution step of 0.5 mm on both principal axes of the plate. The results of the ultrasound imaging given in Figure 4 clearly show the difference between the two plates.

**Figure 5:**
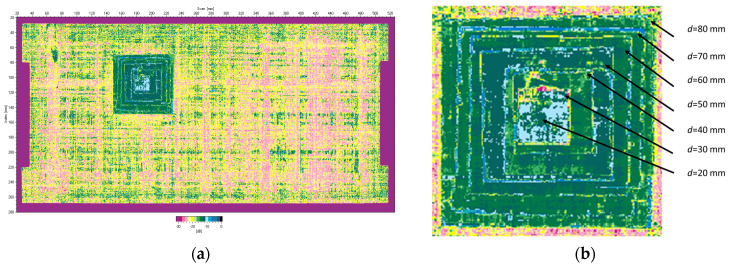
Ultrasonic C-scan (time-of-flight mode) of the plate with artificial damage scanned from the bottom side: (**a**) full scan; (**b**) zoom-in on the delamination.

In Figure 4b, only the largest Teflon insert (*d* = 80 mm) is captured because it is the first limiting surface from which the ultrasonic wave is reflected. The software records this signal as the echo of the defect and also uses it to calculate the depth of the defect. To fully reveal the structure of the introduced delamination damage, the plate was scanned from the opposite side (Figure 5a). The zoomed C-scan image shows the pyramidal structure of the delamination damage and the planar size of each insert that forms it (Figure 5b).

## 3. Development of Optimal Sensor Networks

In this study, the concept and methods for determining the optimal sensor networks presented in [15] are evaluated based on the experimental approach. Furthermore, the analysis of the effectiveness of proposed procedure in the presence of numerous uncertainties, constraints and limitations present in physical SHM systems is investigated.

The optimal sensor network presented in this work was obtained based on the conducted research presented in [15]. During this study, many different methods were tested to determine the optimal sensor network. They include the method based on the absolute strain values calculated for appropriate numerically calculated modes, with each mode having a different weight (method represented as A1), the method based on normalized strains where all modes are equally weighted (method depicted as A2) and the method based on the modified version of an effective independence method (designated as A3) [24,25,26,27]. Each of the methods contains some distance constraints of the distance required between two neighbor sensors and the distance required between the position of sensors and edges of the plate and clamping elements. In the above-mentioned methods, the optimal sensor networks were calculated independently for each direction treating the modal matrix (built on the basis of strain map values for corresponding modes) independently in each direction. Therefore, variants of the methods, called B1, B2 and B3, were also investigated. In these methods, the modal matrix for both directions were treated as a common data set, which may result in a different number of sensors in each of the directions. For detailed information on these methods, developed algorithms and applied constraints, please see Section 3.3 of [15]. All the sensor networks obtained in the mentioned study were developed on the basis of the modal matrix of a calibrated FE model for the healthy condition of the plate only. The modal matrix was obtained as a composition of the strain values in different directions and for seven different modes. The modes were first ordered according to the value of the highest energy modal shape value based on information from the experimental frequency response function.

The concept of the research on the optimal sensor placement was based not only on determining the distribution of the sensor localization, but also on the required number of sensors to be used and the appropriate number of considered mode shapes. For this purpose, many different evaluation functions were used based on the RMS MAC criterion, Fisher matrix determinant and condition number [28,29,30,31]. The aggregation operator of the mentioned evaluation functions, based on the Hamacher function [32], was used in the assessment of sensor networks obtained using different methods, different constraints and different sensor numbers and numbers of modes. The details of this approach and the results are presented in Sections 3.3 and 3.4 in [15].

**Figure 6:**
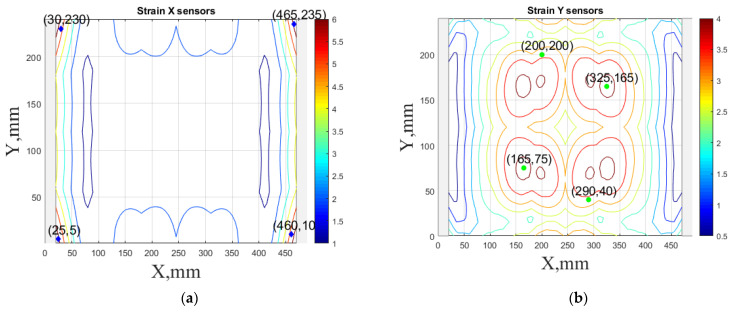
The sensor networks obtained using the A2 method with four sensors in each direction: the sensor network in X (**a**) and Y directions (**b**). Shaded regions represent the mounting clamps.

Finally, this approach was initially evaluated based on a trained, validated and tested set of classifiers prepared on the learning data set. This set was collected on the basis of developed numerical FE models of the plate for healthy and damaged conditions with different localization of the delamination. This localization was not known during the development of the sensor network apart from one sample of data for the healthy condition of the plate. A binary classifier for damage detection and multiclass classifiers for damage localization were used for this initial evaluation (see Sections 4 and 5 of [15]). Performed analysis allows the selection of k-NN classifier as the best performance classifier, robust to noisy data. Additionally, the results of the evaluation of the optimal sensor placement approach indicate that A2 and B2 are the best methods assuming that the number of sensors is not less than four sensors in both directions. These results are presented in Tables 11–14 in [15] and the discussion of these results is presented in Section 5.3 and Section 6 [15]. It is worth mentioning that previously obtained results were evaluated based on numerically derived data and this paper is aimed at the empirical evaluation of the presented approach. For that purpose, results obtained using the A2 method with four sensors in the X direction and four sensors in the Y direction with the application of seventh modes are evaluated with the data-driven experimental approach presented in this paper. This sensor network is presented in Figure 6, where Figure 6a presents the positions of sensors oriented in the X direction and Figure 6 presents the positions of sensors oriented in the Y direction. Dots in Figure 6a (blue) and 6b (green) present the localization of sensors, and the contour lines present the isolines of the sum of absolute normalized strains for seven modes in X (Figure 6a) and Y (Figure 6b) directions, correspondingly.

**Figure 7:**
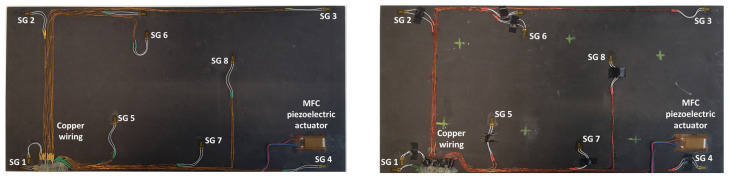
CFRP plates equipped with sensor network. SG refers to strain gauge, and the number corresponds to the sensor number in Table 1.

For the experimental validation of the developed concepts and methods, the CFRP plates were equipped with a strain sensor network (Figure 7) obtained using the A2 method. HBM^®^ 1-CLY41 6/350ZE strain gauges and the MacroFiber Composite™ MFC P1 type piezoelectric actuator were bonded to the surface using Cold Curing Superglue Z70 provided by HBM^®^. The strain gauges were prewired with a 50 mm fluoropolymer-insulated stranded wire by the manufacturer. Copper wires with a cross-sectional area of 0.04 mm^2^ were connected to stranded prewires and glued to the plate surface using the Z70 glue. To minimize the influence of wiring on the experimental results, flexible four-core cables with stranded wires were selected for connecting the sensors to the data acquisition box.

## 4. Preparatory Studies

These preparatory studies present experimental dynamic strain measurements based on the selected sensor network localization on the damaged and undamaged plate, as well as the FE model with piezoelectric transducer preparation and its calibration to these experimental measurements. Both of these steps are used to prepare the modal matrices of the sensor network used in the further evaluation of the proposed sensor network.

### 4.1. Experimental Dynamic Strain Measurements

In the first stage of experimental testing, the acquisition of natural frequencies and corresponding mode shapes of CFRP plates was performed using a Polytec^®^ PSV-500-3D scanning laser vibrometer. For two opposite edges of the plate, the clamped boundary conditions were applied. The clamping was performed using the frame and solid aluminum bar and bolts spaced with a distance of 50 mm and with the fastening torque of 20 Nm. The clamping was performed at a distance of 20 mm from the edge of the plate.

**Figure 8:**
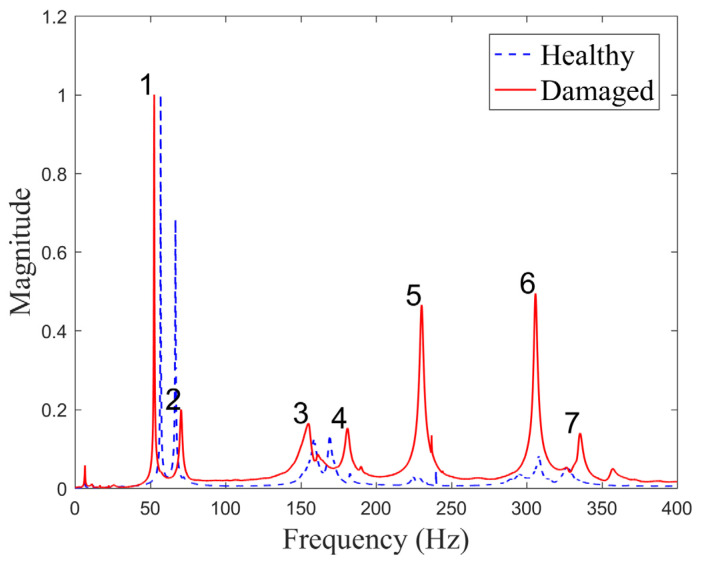
The experimentally measured FRFs for CFRP plates.

The plates were excited via a MacroFiber Composite™ MFC P1 type piezoelectric actuator (with the d33 effect) by introducing a periodic input chirp signal with a bandwidth of 0÷400 Hz bandwidth with a frequency resolution of 0.195 Hz. A 2 V vibration amplitude was generated by the internal generator of the vibrometer and amplified by a factor of 200 using a TREK PA05039 signal amplifier delivered by Smart Materials. The normalized frequency response functions for both plate conditions are shown in Figure 8, while the resonant frequencies are given in Table 2.

The obtained results show that the difference in the measured resonant frequencies varies in the range from −6.9% to 7.5%, suggesting that the introduced delamination has a smaller effect on the stiffness of the plate and, correspondingly, on the frequencies compared to the differences in the plates in general. The plates were produced manually in a scientific laboratory, and, thus, their consistency can be guaranteed only to a certain degree. This also suggests that natural frequencies cannot directly be used for damage identification, and other means of exploiting measured data should be sought for this purpose. Normalized frequency response functions show that the largest vibration magnitude is obtained for the first frequency for both plates. Modes 3 and 4 are in close range for both plates in terms of vibration amplitude, while other modes differ significantly.

For dynamic strain measurements, the plate was excited by a periodic sine wave signal according to the measured resonant frequencies with peak-to-peak vibration amplitudes of 1, 2 and 3 V generated by a waveform generator Agilent 3322A (Agilent Technologies, Santa Clara, CA, USA). Different vibration amplitudes were chosen to evaluate the effect of excitation force on strain measurements and to test the robustness of the damage identification procedure.

**Figure 9:**
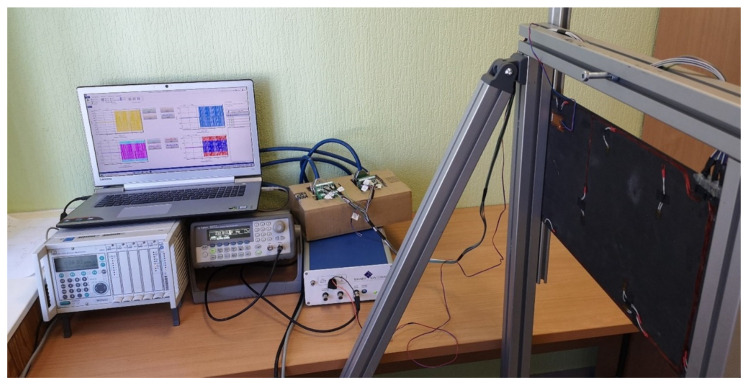
The experimental setup of hardware implementation of the developed SHM system [15].

The strain measurements were recorded using the HBM^®^ MGCplus data acquisition system manufactured by HBM (Darmstadt, Germany) and interpreted by Catman software. The present system (Figure 9) is able to simultaneously record data from eight channels with a maximum sampling frequency of 2400 Hz for each channel. To acquire compatible dynamic strain data, an equal number (4) of sensors was chosen in both principal directions of the plate. Sensors for the network were selected based on the order of the magnitude of the strain values determined by numerical analysis according to the sensor network placement shown in Figure 6. The location of the selected sensors for both plates is given in Table 1.

**Figure 10:**
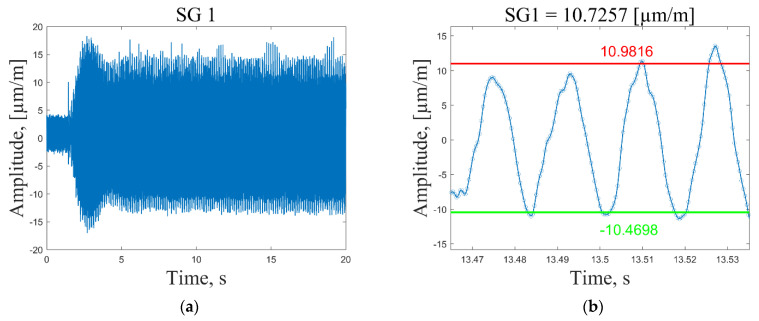
Recorded time series of strain response of the plate excited by a periodic sine wave signal corresponding to the first resonant frequency with a 1 V peak-to-peak vibration amplitude (**a**); calculated average peak-to-peak values for strain gauge 1 (**b**).

**Figure 11:**
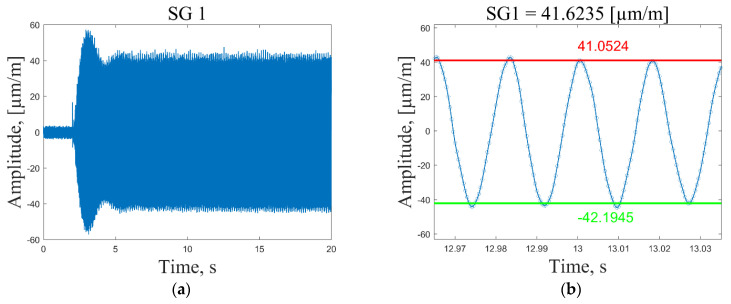
Recorded time series of strain response of the plate excited by a periodic sine wave signal corresponding to the first resonant frequency with a 3 V peak-to-peak vibration amplitude (**a**); calculated average peak-to-peak values for strain gauge 1 (**b**).

**Figure 12:**
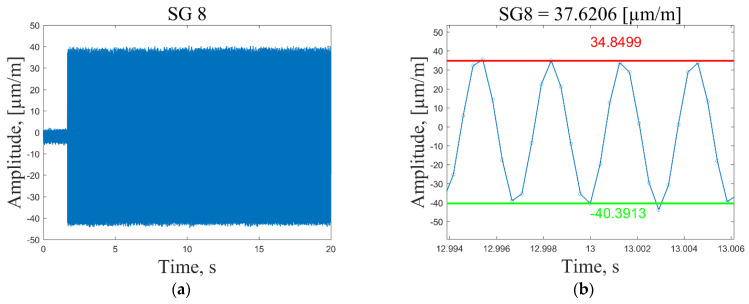
Recorded time series of strain response of the plate excited by a periodic sine wave signal corresponding to the first resonant frequency with a 1 V peak-to-peak vibration amplitude (**a**); calculated average peak-to-peak values for strain gauge 8 (**b**).

**Figure 13:**
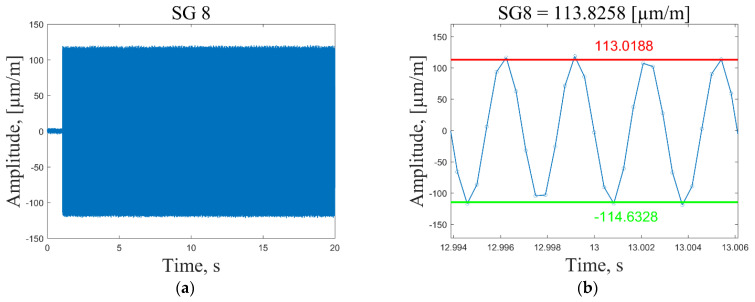
Recorded time series of strain response of the plate excited by a periodic sine wave signal corresponding to the first resonant frequency with a 3 V peak-to-peak vibration amplitude (**a**); calculated average peak-to-peak values for strain gauge 8 (**b**).

In general, the selection of the resonant frequencies for the strain sensing depends on the maximum available sampling frequency of the data acquisition system. To acquire meaningful strain data, one would need at least six data points to draw a corresponding response to the sinusoidal excitation signal. Thus, 2400/6 = 400 Hz is the maximum resonant frequency for which meaningful strain data could be acquired for the present composite plate. Dynamic strain data are collected by following these steps:

1. The data acquisition system is activated and the measurement channels starts recording strain data with a sampling frequency of 2400 Hz (Figure 10, time *t* = 0.2 s).
2. The plate is excited (Figure 10, time *t* = 2 s) by a periodic sine wave signal corresponding to one of the first seven resonant frequencies with a duration of *t* = 20 s.
3. The recorded time series of the strain response are imported into Matlab and the average peak-to-peak strain values are calculated in the period *t* = 8–18 s. Outlier peak values are filtered out using the 2 standard deviation approach. Then, the mean strain value is determined by diving the sum of the absolute average peak values by a factor of 2. For illustration, a recorded strain signal in a very narrow time frame together with the calculated mean and average peak-to-peak values are shown in Figure 10, Figure 11, Figure 12 and Figure 13.
4. The mean strain values for all sensors and resonant frequencies are collected in a matrix of 8 × 7 data points. Calculated strain data are given in Appendix A, Table A1, Table A2 and Table A3, for the undamaged condition of the plate excited using 1, 2 and 3 V vibration amplitudes, respectively. To separate strain data from the effect of the loading conditions and turn them dependent only on the condition of the structure, it is proposed to scale all sensor measurements with respect to the strain value of the first sensor in a considered direction.

In Figure 10a and Figure 11a, one can see that at the excitation moment (time *t* = 2 s) there is a large spike in the strain plot, which decays in a few seconds and then the signal normalizes for the rest of the measurement time. This effect is observed only for the first resonant frequency, while for the other modes, strain measurements are in the range of peak values starting from the excitation moment (Figure 12a and Figure 13a). However, it should be noted that average peak-to-peak strain values are calculated in the period *t* = 8–18 s; thus, these disturbances do not affect the calculated values. Figure 11b shows a smooth sinusoidal response of strain gauge 1 to the 3 V excitation amplitude of the plate corresponding to the first resonant frequency.

All recorded peak values (both positive and negative) are in close proximity to the calculated average peak-to-peak strain values. In turn, in Figure 10b, one can see that in this narrow time frame, some recorded strain values differ significantly from the calculated average peak-to-peak strain values. This is explained by the fact that, by using the peak-to-peak vibration amplitude of 1 V, the recorded strain values are also significantly lower (three times) compared to the ones obtained by the 3 V excitation amplitude, and are, therefore, influenced by the measurement noise to a greater extent. The obtained results showed that for low strain amplitudes (below 15 µm/m), the recorded signal is inconsistent. For this reason, the 2 instead of the 3 standard deviation approach was adopted for the removal of outliers. To provide the same preprocessing conditions for every excitation signal and amplitude, it was decided to employ the 2 standard deviation approach for signal conditioning and acquisition of peak strain values in all cases. It must be noted that in the time frame used for the calculation of average strain values, there are 600 positive and 600 negative peaks for the first resonant frequency, which allows one to obtain reasonably good average strain values.

### 4.2. Evaluation of Experimental Data

To evaluate the consistency of the experimental strain data, statistical methods for the comparison and quantitative correlation between the measured dynamic properties were looked up. One of the most popular tools for quantifying modal vectors is the Modal Assurance Criterion (MAC). MAC is a statistical indicator, similar to the coherence feature, which reveals a sensitivity to a difference between compared values, and similarly remains relatively insensitive to small changes or small magnitudes. This gives a good statistical indicator and a degree of consistency between the compared mode shapes and its effectiveness is confirmed in practical applications (see, e.g., [33]). It is limited between 0 and 1, with 1 showing completely consistent mode forms. It can only show consistency and does not show validity or orthogonality. A value close to 0 indicates that the modes are not consistent [34].

**Figure 14:**
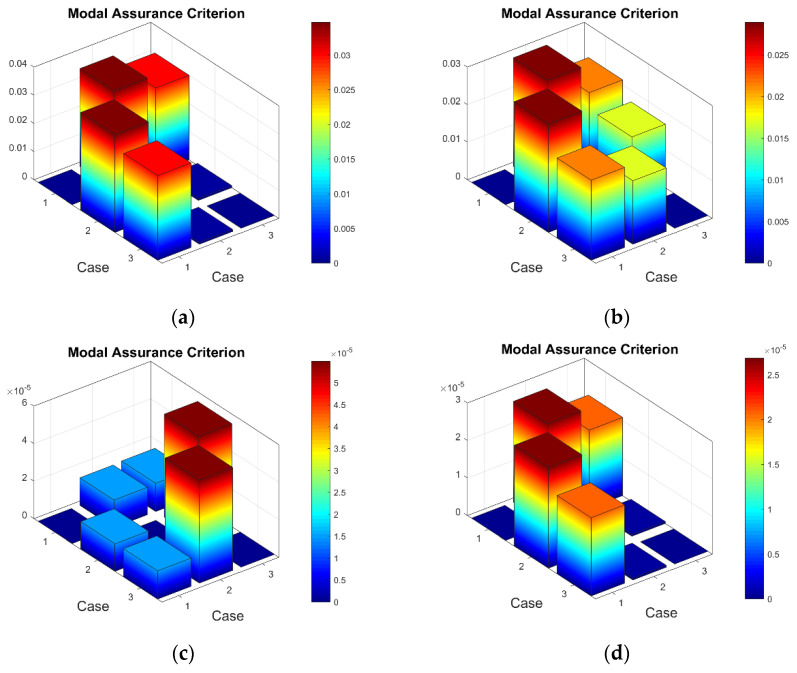
The selected MAC values for strain data obtained using 1, 2 and 3 V vibration amplitudes: (**a**) healthy plate for the first resonant frequency, (**b**) damaged plate for the first resonant frequency, (**c**) healthy plate for the seventh resonant frequency, (**d**) damaged plate for the seventh resonant frequency.

Firstly, it was of interest to compare the consistency of the measured strain data of the test plates excited by different vibration amplitudes (Appendix A, Table A1, Table A2 and Table A3). It must be noted that calculated and then scaled mean strain data for eight sensors are treated as the mode shape of a particular resonant frequency for the calculation of the MAC. The 3D MAC plots for healthy and damaged plates are given in Figure 14.

For the record, in Figure 14, Case 1 corresponds to the mode shape obtained by a vibration amplitude of 1 V, and Cases 2 and 3 correspond to vibration amplitudes of 2 V and 3 V, respectively. One can see that for the first resonant frequency, there is a slight deviation between the mode shape obtained using the 1 V excitation amplitude and the mode shapes acquired using 2 and 3 V (the maximum ‘1-MAC’ value is 0.04). In turn, in Figure 14, the MAC values are close to 1 for the mode shapes of the seventh resonant frequency, showing good consistency between them (the maximum ‘1-MAC’ value being 6 × 10^−5^). Similar MAC results are also obtained for mode shapes of the third to sixth resonant frequencies. It can be explained by the fact that the strain values (in µm/m) obtained for the first two frequencies of the plates excited by the 1 V peak-to-peak vibration amplitude are comparatively lower than for the higher resonant frequencies and are, thus, affected by the measurement noise to a greater extent.

**Figure 15:**
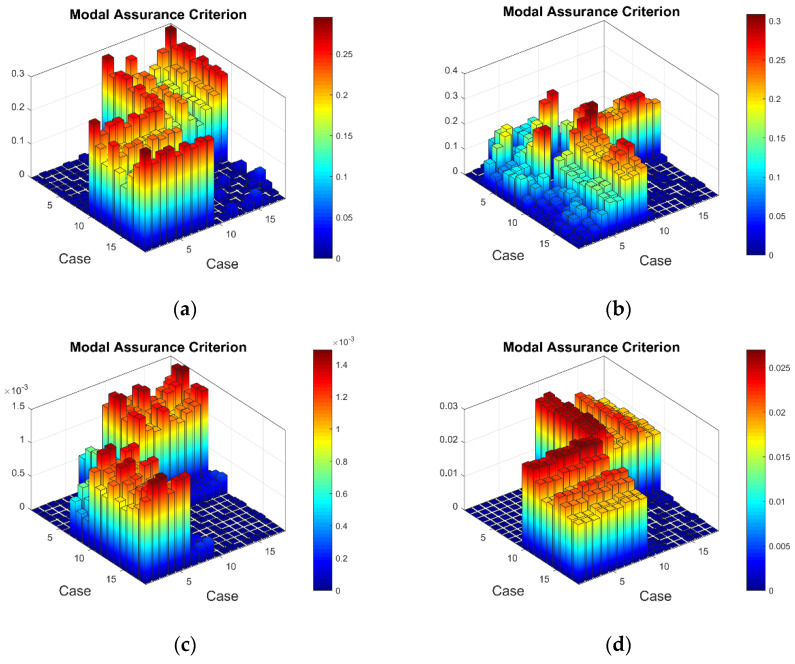
The MAC values for 18 sets of strain data: (**a**) healthy plate for the second resonant frequency, (**b**) damaged plate for the second resonant frequency, (**c**) healthy plate for the fifth resonant frequency, (**d**) damaged plate for the fifth resonant frequency.

To evaluate the consistency and performance of sensors and experimental equipment, as well as the effect of environmental conditions (changes in room temperature and humidity) on strain measurements, additional tests were performed. Within two weeks, 3 × 3 sets of strain data were acquired for both healthy and damaged plates. Furthermore, the influence of boundary conditions on sensor data was tested by removing the plates from the test stand and setting up the experiment once more following the same procedure as before. Again, 3 × 3 sets of strain data were obtained for both plates within the span of two weeks. Thus, a total of 18 sets of strain data were acquired for the first seven resonant frequencies of the healthy plate and the same amount of data for the damaged plate. MAC results indicated that the performance of the experimental equipment is consistent and that the effect of environmental conditions on the strain measurements is minimal. On the other hand, the effect of boundary conditions (replication of the experimental setup between two tests with clamped boundary conditions can be guaranteed only to a certain degree) is evident, although not to a large extent. Details about the MAC results comparing strain data obtained between two experimental setups are shown in Appendix B. Selected MAC plots for the entire set (in total, 18 sets comprising 9 sets for each experimental setup) of the strain measurement data are shown in Figure 15.

### 4.3. Finite Element Model with Piezoelectric Transducers

The development of the proposed data-driven monitoring system requires the preparation of a data set which contains strain values for the localizations related to the selected optimized sensor network. To reduce the number of experiments that allow one to gather experimental strain data, numerical experiments are also conducted. In this case, appropriately prepared and tuned FEM models, which are presented in this section, are used to perform numerical simulations to obtain the learning data set. This data set is used mainly for training and validation, but sometimes also for initial testing of the data-driven system response, excluding experimental validation cases. In this approach, these learning data sets are intentionally noisy in order to obtain a unique set of learning samples and to develop the initial validation of the structural monitoring system.

The development of the plate FE model was performed using material properties (staking sequence, material constants) obtained in the previous study [15]. The geometric model of the plate was meshed with SOLSH190 elements and a regular mesh of 90 × 48 elements was obtained. Two opposite edges of the plate were fixed according to the scheme presented in Figure 1. A single element in the thickness direction was attributed to each layer of a composite, giving a total 20 elements for the entire composite.

For supervised learning oriented to damage detection, an FE analysis of the developed model was performed. The strain values were acquired in both principal directions of the plate at the predefined sensor locations (Table 1) in the form of a vector of 56 data points (seven modes × eight sensors). The class label of ‘0’ was assigned to the vector representing the undamaged condition of the plate. Further, damage in the form of delamination specific to the low-velocity impact of a composite plate was implemented in the FE model. To appropriately train the classifier models, both the dimensions and location of the simulated delamination were subject to change. The investigation of a dynamic response of the tested composite structures for the damage scenarios considered in this study was based on the experiment plan presented in Table 3. The considered damage scenarios were represented by the center point coordinates (*dx_c_* and *dy_c_*) of the simulated delamination. The variation in dimensions and location of simulated delamination *d* is represented by the assumed scheme (see Figure 1). The presence of delamination in the numerical model was introduced by contact deactivation in particular nodes within the delamination area defined by a given scenario. In total, 288 FE models have been developed to determine the strain values in both main directions of the plate at predefined sensor positions (Table 1) for the first seven resonance frequencies. In this way, a matrix of 288 × 56 data points is obtained for the training of machine learning algorithms.

Within the learning scheme that focuses on the detection of damage, the assignments of class labels were performed. For all 288 data vectors, which represented the plate with delamination damage, class label ‘1’was assigned. Additionally, for the learning scheme used for localization of damage, four class labels (from ‘1’ to ‘4’) were assigned according to the four substructures defined in Figure 1, which correspond to the coordinates of a center point of delamination according to Table 3. As a result of the performed FE simulations, 289 and 288 sample points were obtained for the damage detection and localization learning schemes, respectively. The number of sample points in the first case represents one case for an undamaged plate and 288 cases for a plate with simulated delamination with various geometric parameters. In the second case, the number of sample points represents 72 data vectors for each of the four class labels describing the defined substructures of the location of the delamination in the plate. Taking into account the fact that experimental testing involves an MFC actuator for vibration excitation, in this investigation, harmonic analysis was considered to determine the eigenvalues and corresponding eigenvectors instead of the modal analysis employed for FE model performed on numerical results in the previous study [15].

**Figure 16:**
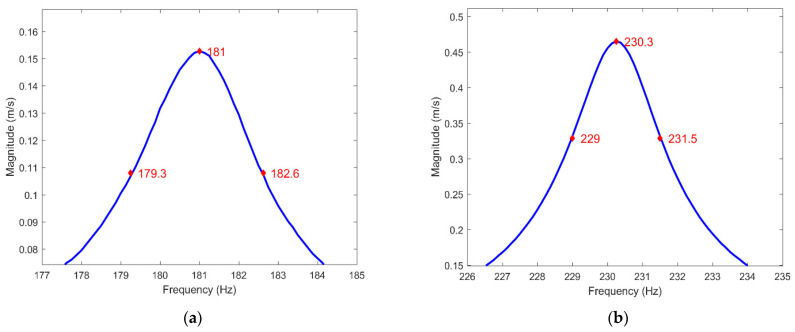
The half-bandwidth method for modal loss factor extraction for (**a**) the fourth mode; (**b**) the fifth mode (see Table 2 for details).

To perform harmonic FE analysis, in addition to elastic constants, damping properties of CFRP plates are also necessary. For this reason, the experimentally obtained FRF of the CFRP plates was used for the extraction of the modal loss factors by employing the half-bandwidth method. The experimental modal loss factors were assessed based on the frequency-domain single degree of freedom (SDOF) principle. This principle introduced a sequential evaluation in the vicinity of each peak of the FRF modulus plot, which can be represented by the peak amplitude method (see [35] for more details). According to this method, the following formula can be used to determine the modal loss factor $\eta_{n}\;$extracted from the experimental FRF for a given resonance peak:

(1) $$\eta_{n}=\frac{f_{n2}-f_{n1}}{f_{n}},$$

where *n* is the mode number; *f_n_*_1_ and *f_n_*_2_ are the frequencies whose values were interpolated from the FRF modulus plot. Illustration of the method is given by the exemplary resonant frequencies in Figure 16 and Appendix C. The calculated modal loss factors for the considered resonant frequencies are given in Table 4. An average modal loss factor was used for harmonic analysis to obtain numerical FRFs of the CFRP plate in the frequency range from 0 to 400 Hz with a frequency resolution of 0.25 Hz.

**Figure 17:**
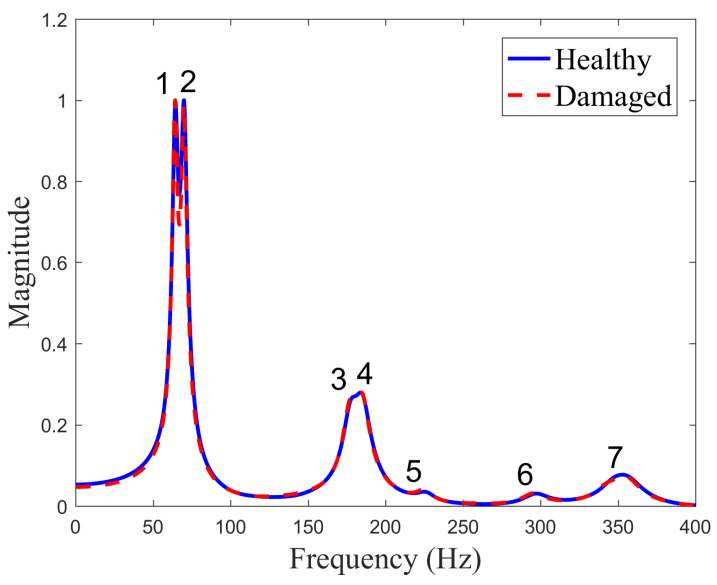
The numerically calculated FRF of CFRP plates.

The MFC actuator patch with a size of 30 × 15 mm and a thickness of 0.3 mm was bonded to the surface of the plate according to the scheme shown in Figure 1. The MFC actuator was modeled with 20-node coupled-field quadratic brick SOLID226 elements. The material properties used for the development of a linear electromechanically coupled finite element model of MFC actuator are given in Table 5, where *E*_33_ and *E*_11_ are the tensile moduli in the rod and electrode directions, respectively; *G*_31_ is the shear modulus; *ν*_31_ and *ν*_13_ are the Poisson ratios with respect to the material coordinate system; *d_33_* and *d_32_* are the strains per unit electric field in the rod and electrode directions, respectively; and *ε^T^_11_*, *ε^T^_22_* and *ε^T^_33_* are the relative permittivity with respect to the material coordinate system. For harmonic analysis, the MFC patch is actuated by a drive voltage of 400 V. The calculated frequency response functions for both plates conditions are shown in Figure 17 with resonant frequencies given in Table 6.

One can see that the difference in plots of FRFs is barely visible and changes in numerical values of resonant frequencies are in the range of −0.6 to 0.6%. Similar to experimental measurements, this indicates that changes in resonant frequencies may not be effectively used for damage identification in the present investigation, and other means of exploiting modal data should be sought for this purpose.

### 4.4. Finite Element Data Evaluation and Preprocessing

In total, 289 sample points (1 for the healthy condition and 288 for the damaged condition) were obtained from the FE harmonic analysis. Each sample point consists of a data vector of 56 sensor measurements, i.e., for each of the seven considered modes within the FE simulation results, 8 sensor measurements were noted. Due to large differences in strain magnitudes acquired from strain sensors, a normalization was applied to avoid a possible problem of ill-conditioning. The normalization procedure was carried out as follows: the first sensor strain in a specific direction was considered as a reference to which all the rest values were normalized. It is especially important to appropriately train classifiers for discriminant features that are not related to loading and environmental conditions, while remaining dependent on the structural condition only. The goal of the present investigation is to build classification models on the strain data acquired from the FE analysis and then use them for the experimental damage identification in composite plates.

**Figure 18:**
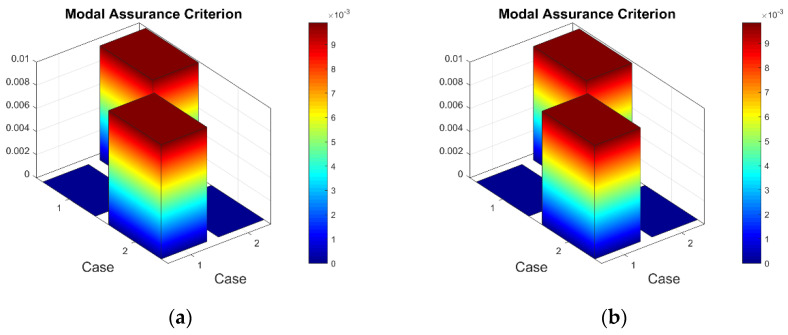
The MAC values between two conditions of the plate: (**a**) first resonant frequency, (**b**) seventh resonant frequency.

**Figure 19:**
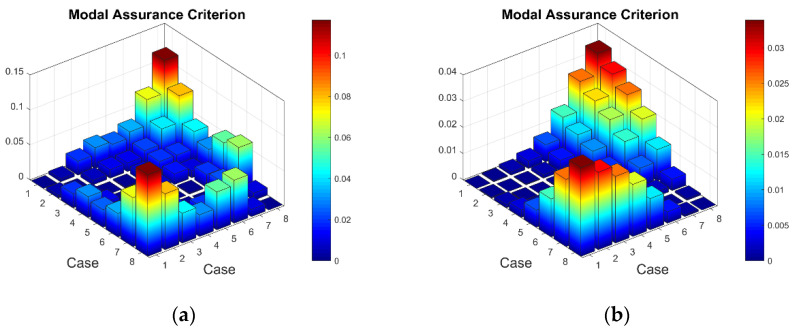
The MAC values comparing strain mode shapes for different location of delamination damage: (**a**) first resonant frequency, (**b**) seventh resonant frequency.

Now when strain data is normalized, it was of interest to evaluate obtained data by statistical methods for the comparison and quantitative correlation. Firstly, it was proposed to compare the mode shapes of the strain data between two conditions of the plate. The 3D plots of calculated MAC values for the first and seventh resonant frequencies are given in Figure 18. The obtained MAC values range from 0.981 to 0.99 depending on a particular frequency, which corresponds to 1–2% changes and is up to four times higher than changes in resonant frequencies. Next, of interest was to evaluate the influence of the damage location on the strain data. In Figure 19, the calculated MAC values comparing strain mode shapes between eight different damage scenarios (delamination location: *d_xc_* = 75 mm, *d_yc_* = 45...105 belongs to the substructure ‘1’ of the plate in all cases) are depicted. One can see that changes in strain data vectors increase gradually as the location of damage increases from the first to the last considered damage scenario. The obtained ‘1-MAC’ values range from 0.12 to 0.04 depending on the distance between the center point coordinates of different delamination damage scenarios.

## 5. Results on Structural Health Evaluation

The application of the developed data-driven structural health evaluation method is demonstrated experimentally on CFRP rectangular plates. The k-NN machine-learning algorithm within two supervised learning schemes is used to create classification models by learning from the simulated response data. The first learning scheme (oriented to damage detection) involves the development of a binary classifier model that sets a description of normality. This represents healthy conditions and abnormality indicating the presence of delamination in a composite structure. The learning data within the damage localization learning scheme additionally contain the known class labels pointing to the damage location. In both learning schemes, the outputs of the binary classifiers were as follows. In the damage detection learning scheme, one obtains a discrete class label representing the structural condition (healthy or damaged), while in the damage localization learning scheme, the result of classification is represented by a discrete class label describing spatial location of damage. The predictive performance of the developed classification models is evaluated by introducing the trained models to unseen experimental data and performing the damage identification on composite plates based on established locations of sensors.

### 5.1. Training Dataset

In the first learning scheme, the following procedure is implemented:

- Strain sensor readings for damaged cases represented by 288 data vectors were replicated ten times (with added noise according to the next step of data preparation), resulting in 2880 data vectors;
- The replication factor of 2880 was applied to the data vector of strain sensor readings for the undamaged composite structure. This implied obtaining 2880 identical sample points for the healthy condition;
- Data vectors replicated in the above readings for damaged and undamaged conditions were made noisy by the addition of 1% noise [15]:

(2) $$s=\overset{´}{s}\left(1+\delta \left(2r-1\right)\right)$$

where $\overset{´}{s}\;$is a noise-free data point, *r* is the uniformly distributed random values in the interval [0,1] and *δ* is the noise level.

- Consequently, a total of 5760 balanced sample points (2880 samples each of both healthy and damaged conditions on the plate) are obtained for the training data;
- The testing data contains two equal groups of 576 sample points: the first group contains 288 different sample points for the damaged condition of a structure, while the second group contains 288 replications of a single sample point for the healthy condition.

The data for the second learning scheme were prepared as follows. For testing purposes, only the original 288 sample points representing the damaged condition of the plate were considered, while for training, 2800 sample points were used. These sample points were obtained from the FE data by adding additional noise at the level of 1%. Due to this, it was possible to reach a situation where there were no identical sample points between training and testing datasets, so these are not used for classifiers training.

### 5.2. Training and Classification Validation Procedure

**Figure 20:**
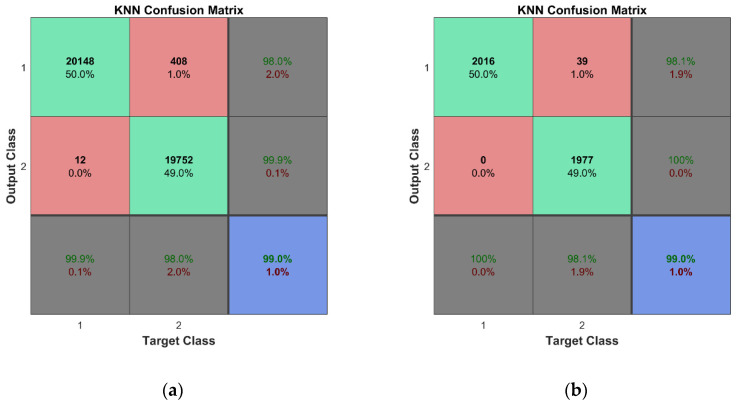
Classification results for the first learning scheme: (**a**) cross-validation results; (**b**) predictive performance on unseen FEM data.

**Figure 21:**
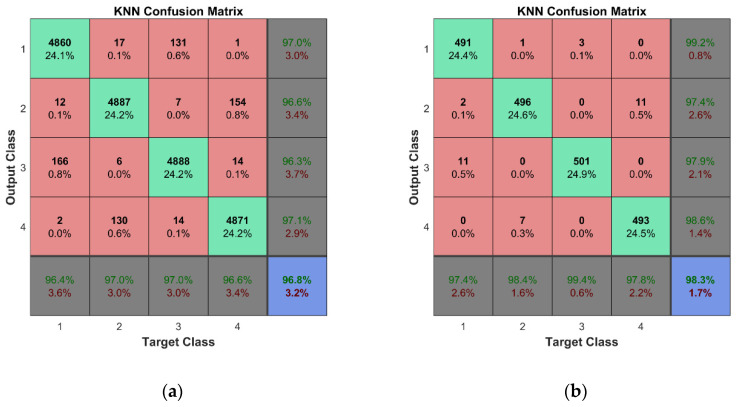
Classification results for the second learning scheme: (**a**) cross-validation results; (**b**) predictive performance on unseen FEM data.

For training and cross-validation, a 10-fold cross-validation scheme (described in [37]) was used. Fine classification results for both learning schemes assumed in this study were obtained thanks to the large set of training data, 10-fold cross-validation, and fine adjustment of the classifiers’ hyperparameters. In the case of the k-NN algorithm, two of the most important hyperparameters are the number of nearest neighbors and the distance metric. The k-fold cross-validation is used to evaluate the performance of different combinations of these hyperparameters and the best one is selected based on the highest average performance across all k folds. Therefore, the combination of the hyperparameters is different for each of the trained k-NN classifiers. This technique helps to ensure that the hyperparameters of the k-NN algorithm are selected in a rigorous and systematic manner, leading to better results and a more robust model. The illustration of the technique implemented in Matlab is given in Appendix D. Table A4 and Figure A6 and Figure A7 show the hyperparameter optimization results for the k-NN classifier trained for the first mode of the second learning scheme. The best combination of the hyperparameters for the particular classifier model is as follows: the number of nearest neighbors—4; the distance metric—‘Minkowski’. The selected number of objective function evaluations (30 in this case) was sufficient to find the best combinations of hyperparameters for all trained classifiers. One should note that classifiers were trained separately for each mode; therefore, seven classification models have been created for both the considered learning schemes. According to this, we obtained a much lower probability of accidental erroneous classification, since most of the obtained results confirmed estimated class of observations, and the possibility of choosing modes with the best overall accuracy was reached with avoiding the modes with unsatisfactory classification results if this was confirmed by more than one classifier. An overall classification accuracy of 99% is observed for binary classifier models, while an 96.8% overall accuracy is obtained for multiclass classifier models. For the representation of the cross-validation of the classifiers, the confusion matrices for all seven classification models were summed in one plot (Figure 20a and Figure 21a). Thus, a total number of 2 × 2880 × 7 class estimates is observed for the first learning scheme while 4 × 720 × 7 class estimates are observed for the second learning scheme.

The cross-validation of the developed models was followed by the testing of predictive performance of classification models on unseen FE strain data—the testing data sets not used for the training of the classifiers. For the binary learning scheme, the classifiers were examined using the unseen testing data comprising 288 cases for both conditions of the plate. The classification results are presented by a confusion matrix containing a total of 2 × 288 × 7 observed class estimates (Figure 20b). The overall accuracy of 99% indicates the effectiveness and robustness of the k-NN classifier for this task. For the second learning scheme, the predictive performance of the classifiers was assessed based on unseen testing data obtained from FEM simulations, which included 288 cases (72 data vectors for every class label representing four substructures) for the damaged condition of the plate. The overall classification accuracy for the seven classifier models is presented in Figure 21b. The k-NN algorithm again demonstrated outstanding performance—the classifier model can accomplish this task with a total accuracy of 98.3%.

### 5.3. Results of Classification for Experimental Data

The experimental investigation is concluded by examining the predictive performance of the built classifiers in Section 5.2 on unseen experimental strain data.

#### 5.3.1. Damage Detection

The unseen experimental testing data, which contained 18 cases that represent both healthy and damaged plate conditions in the first learning scheme, were used for the examination of the trained classifiers by applying 10-fold cross-validation. The predictive performance of the k-NN classifier built for each of the seven considered modes indicated that the poor performance of damaged cases was properly recognized with many false alarm cases. The explanation can be observed by comparing the experimental and numerical strain vectors for the undamaged and damaged cases. The changes in strain vectors between healthy and damaged conditions in the case of FE analysis are relatively small (Figure 18). Thus, the classifier models are built using the data where even the slightest deviation from normality is classified as damage.

**Figure 22:**
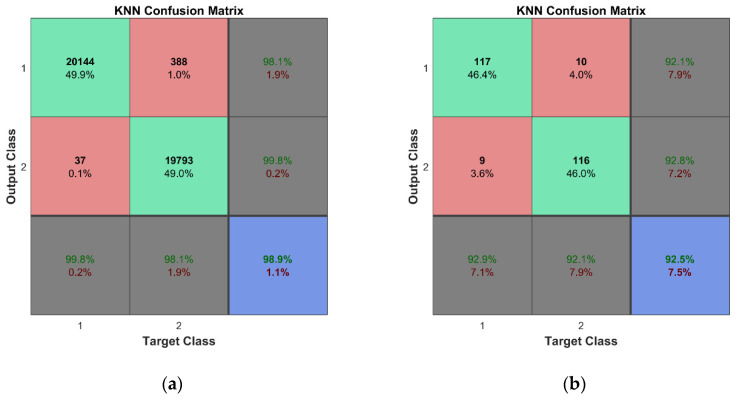
Classification results for the first learning scheme: (**a**) cross-validation results; (**b**) predictive performance on unseen experimental excluding three sets of experimental data that are included.

**Figure A1:**
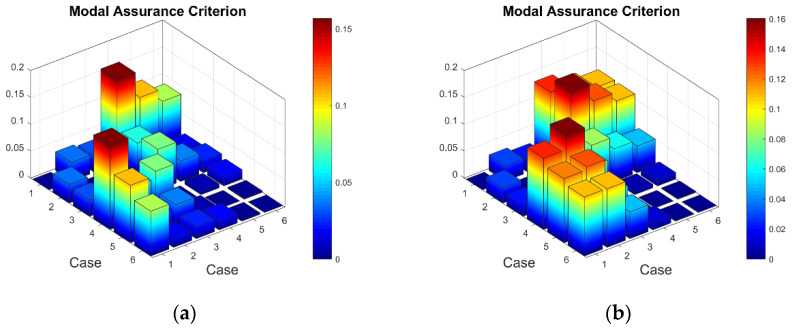

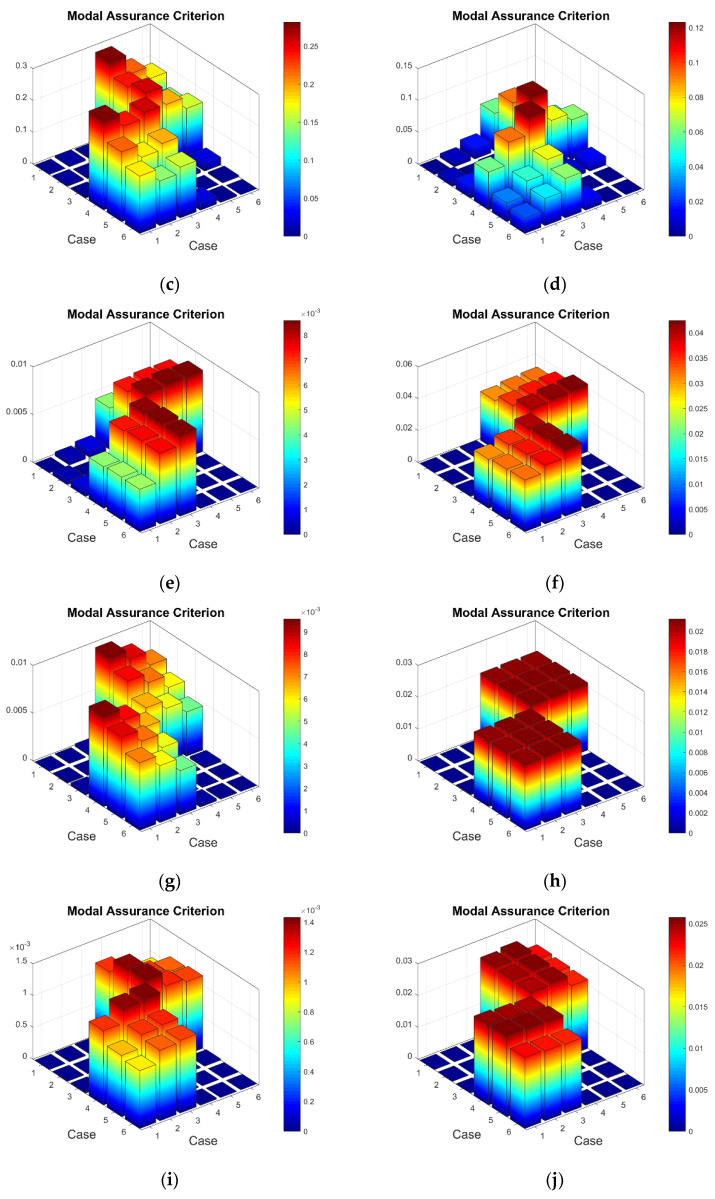

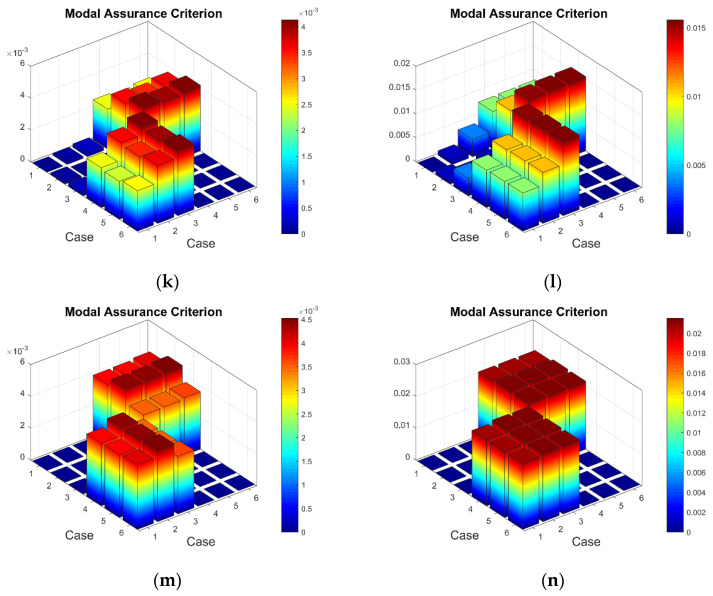
The MAC values comparing strain data between two experimental setups for subsequent frequencies for (**a**,**c**,**e**,**g**,**i**,**k**,**m**) the healthy plate and (**b**,**d**,**f**,**h**,**j**,**l**,**n**) the damaged plate.

**Figure A6:**
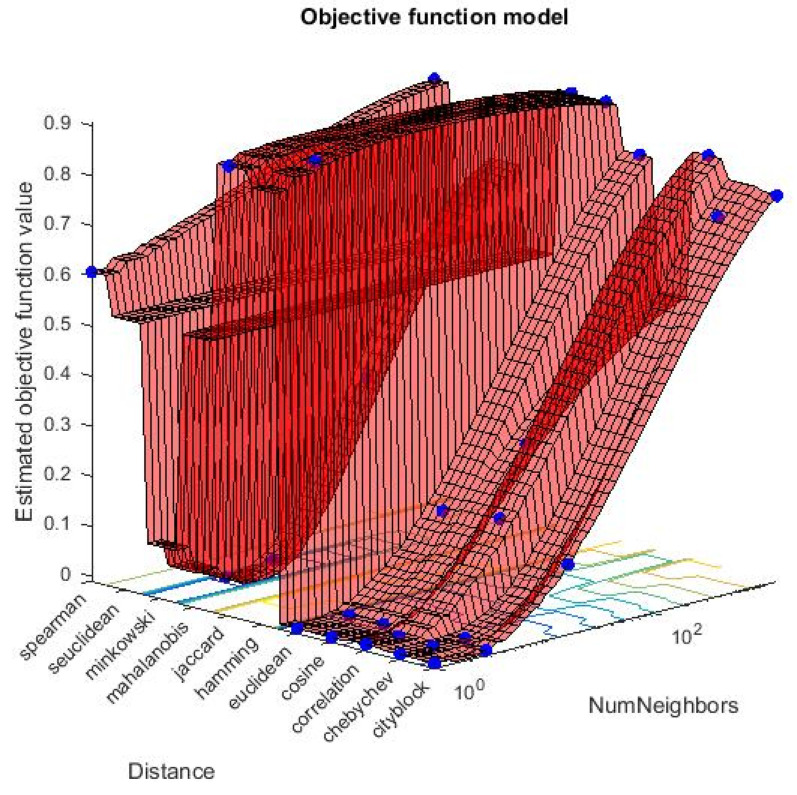
Objective function model for the k-NN classifier trained for the first mode of the second learning scheme (higher precision of localization).

**Figure A7:**
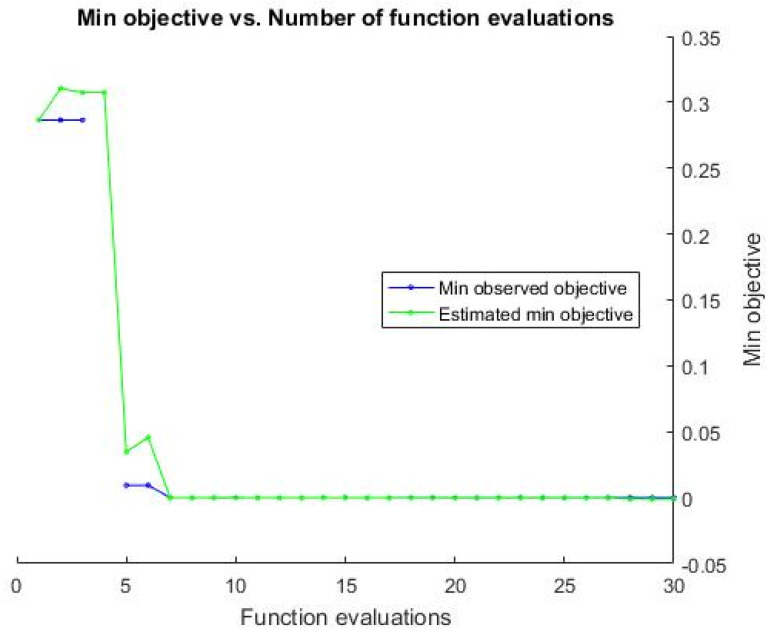
Objective function evaluation plot for the k-NN classifier trained for the first mode of the second learning scheme (higher precision of localization).

To enhance the performance of the classification models, the addition of experimental data sets to the training data was proposed. For this reason, in the first stage, three sets of experimental data out of 18 available experimentally measured strain data vectors biased with a noise level of 1% and included in the training data (strain measurement data obtained for both conditions of the plate excited with 1, 2 and 3 V vibration amplitudes). This leads to a considerably high predictive performance (92.5%) of the classification models (see Figure 22). By examining the performance of classifiers separately, one can see in Table 7 that six out of seven classifications models have correctly distinguished the damaged condition of the plate from the undamaged one. The explanation of the poor performance of the classification model for the second resonant frequency can be found in Figure 16 and Figure A1 (Appendix B). One can see that the mode shapes of the second resonant frequency are the least consistent between different experimental setups and excitation amplitudes. It must also be noted that the added experimental sets are polluted with a significant noise level, and are, thus, not identical to the unseen experiment data used for the testing of classification models. Additionally, three sets of added noisy experimental data comprise only 0.1% of the total number of training data.

#### 5.3.2. Damage Localization

**Figure 23:**
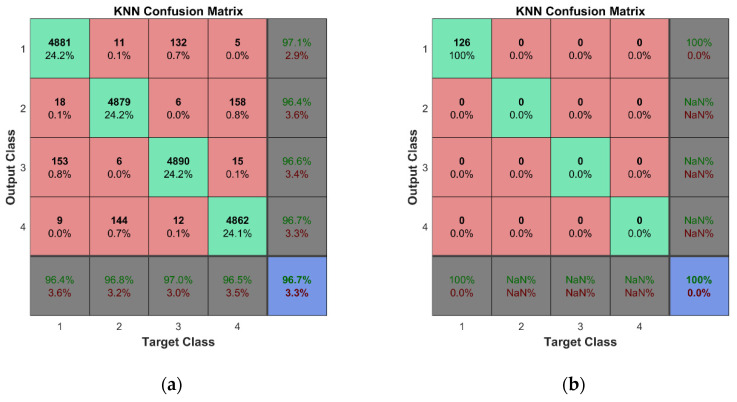
Classification results for the second learning scheme: (**a**) cross-validation results; (**b**) predictive performance on unseen experimental data.

For the second learning scheme, the classifiers trained via 10-fold cross-validation are examined using experimental testing data and three sets of experimental data with added noise out of 18 sets. A class label of ‘1’ is assigned to all 18 vectors corresponding to the location of the delamination damage according to the scheme given in Figure 1. The classification results for all classifiers are summarized in one confusion matrix depicted in Figure 23. The results show very good accuracy in recognizing damage localization.

#### 5.3.3. Damage Localization with Refined Division

**Figure 24:**
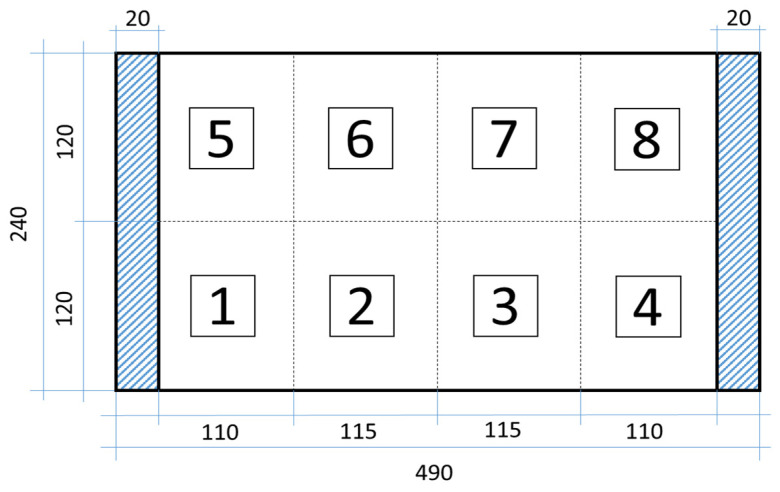
CFRP plate with substructures for damage localization.

Particular attention should be applied to maintaining a degree of rational precision of damage localization, i.e., adequate data granulation [38]. For that purpose, an additional performance of the analyzed damage localization was performed with the refine division of the plate in substructures pointing to the geometrical location of the damage. In this case study, the plate is divided into eight imaginary zones according to the scheme depicted in Figure 24.

In total, 288 FE models with artificial delamination damage were built and harmonic analyses were performed for the second learning scheme. Class labels ‘1’ to ‘8’ are designated for each of the 288 data vectors representing the damaged condition of the plate corresponding to one of the eight substructures in which the center point of delamination is located (Table 3). Based on this information (the location of the center point of the delamination), 32 data vectors belonging to zones ‘1’, ‘4’, ‘5’ and ‘8’ were assigned, while 40 vectors belonged to zones ‘2’, ‘3’, ‘6’ and ‘7’. Training and testing of the machine learning algorithms was carried out according to the procedure described in Section 5.1 and Section 5.2. The testing data include only the 288 original samples for the damaged condition of the plate, while training data of 2880 points are derived from the FE data using the multiplication factor of 10 and a noise level of 1%. A class label of ‘2’ is assigned to all 18 experimental data sets that correspond to the location of delamination damage according to the scheme given in Figure 24. A 10-fold cross-validation scheme is used for the training and cross-validation of classification models. When testing using experimental data with the incorporation of the three sets of experimental data with added noise to the learning dataset, the overall classification accuracy reached 98.4%. This result indicates that the classification accuracy is still high and allows one to maintain a rational precision degree of damage localization.

Table 8 contains a summary of results obtained in the presented research. Every time the training data contains experimental data, the fraction of experimental data is 0.1% in the training data set.

### 5.4. Practical Implications

In practice, the developed structural health evaluation method may be applicable for composite structural components that are produced in large quantities with a high degree of replicability and used for designed purposes, for instance rotor blades. In this case, costs of development and calibration of a high-fidelity FE model would be justified since one model is valid for millions of produced copies. Additionally, for such a structural component, a designer of the SHM system can easily define the potential failure modes, their possible locations in structure, and which of these modes are crucial. This information may then be used to implement possible damage scenarios in an FE model. Defined damage thresholds allow us to determine if structural component has to be repaired or replaced according to the online strain measurements.

For the training of the classification algorithm, the addition of experimental data is suggested as it may improve the predictive performance of the classifier, as is evident in this study. For the structural component manufactured on an industrial scale, few experimental tests would still be cost-effective, taking into account the possible benefits of the integrated SHM system. The benefits of a permanent and independent SHM system integrated within a structural component include optimum utilization of the structural component, reduced maintenance costs, minimization of downtime and avoiding catastrophic failure. Independent SHM systems will increase the operational safety of structural components, give them a technological added value and extend the areas of their application.

## 6. Conclusions

The paper presents the application of the developed data-driven structural health evaluation method for the identification of experimental damage in CFRP plates. The k-NN machine learning algorithm within two supervised learning schemes is used to create classification models by learning from simulated response data and three sets of experimental data with added noise. The predictive performance of the developed classification models is evaluated to unseen experimental data and is used to identify damage in composite plates. The classification process is performed on strain values from optimal sensor placement localizations obtained using method A2 and the methodology presented in [15].

The first learning scheme involves the development of the binary classifier models which were used for damage detection to indicate the presence of delamination in a composite structure. In the second learning scheme, the learning data representing the damaged condition of the structure, also as depicted as class labels, points to the geometrical location of the damage. The trained k-NN classifiers yielded an overall classification accuracy of 99% and 98.3% for the respective learning schemes for unseen numerical strain data, thus providing theoretical confirmation of the effectiveness and robustness of the algorithm for both damage detection and localization tasks. To enhance the generalization properties of the obtained classifiers for prediction of the classification models, the addition of experimental data sets with added noise to the training data was proposed. The obtained results show that by adding three sets of measurement data, the overall classification accuracy increases to 92.5% for the binary classification and maintains this value for the multiclass classification, including detection and localization. High accuracy is also maintained for a higher degree of damage localization, resulting in 91% for multiclass classification, including detection and localization.

The results show that although the k-NN machine learning algorithm theoretically performs classification tasks with very high accuracy, in practice, strain data is corrupted by measurement noise, affected by changing environmental conditions, etc., and its effectiveness reduces significantly. To overcome this problem, data that combine numerical and experimental monitoring data with added noise are proposed. By combining features extracted from different sensor measurements and integrating them into a single feature, it was shown that structural health evaluation performance is significantly enhanced.

In summary, the following conclusions can be drawn:

(a) Measurement noise, environmental conditions and boundary conditions strongly reduce the effectiveness of damage detection and localization using classifiers when they are trained using only artificial data from numerical simulation.
(b) A high accuracy of delamination detection and localization in composite plates can be obtained by adding a small amount of the learning examples from the experimental part (in the considered case, it is only 0.1% of the total number of training data). These examples include the changing of boundary conditions and are affected by measurement noise.
(c) In the case of a higher accuracy of the damage localization, a higher precision of the damage localization can be the subject of optimization. The objective function can be customized to achieve a trade-off balance between the localization precision and accuracy of the damage classification.
(d) The proposed approach should be preceded by the reliable application of optimal sensor network selection with a reduced number of sensors (proposed also by the authors) and the selection of an appropriate classifier based on its predictive performance.

The presented approach demonstrated great performance in identification damage with a limited number of sensors, which also justifies its economical effectiveness, especially when large spatial structures are the subject of monitoring. Additionally, the robustness to measurement noise and changes in boundary conditions, together with the practical implications mentioned in Section 5.4, justify its practical applicability.

## Figures and Tables

**Table 1:** The configuration of the sensor network for the dynamic strain measurements.

Sensor #	1	2	3	4	5	6	7	8
*x*, mm	25	30	465	460	165	200	290	325
*y*, mm	5	230	235	10	75	200	40	165
orientation	0^°^	0^°^	0^°^	0^°^	90^°^	90^°^	90^°^	90^°^

**Table 2:** The experimental resonant frequencies (Hz) and corresponding modes.

No.	Mode	Healthy	Damaged	Δ
1	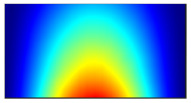	56.9	52.6	7.5
2	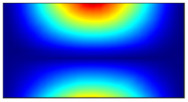	66.8	70.5	−5.5
3	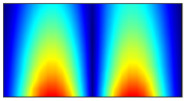	158.5	155.1	2.1
4	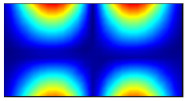	169.3	181.0	−6.9
5	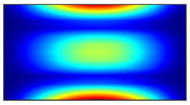	224	230.3	−2.8
6	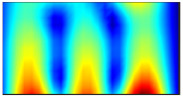	308.1	305.9	0.7
7	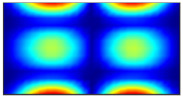	327.1	335.5	−2.6

**Table 3:** The experimental plan for the considered damage scenarios.

No.	*dx_c_*	*dy_c_*	Class
1	75	45	1
2	75	55	1
…			
9	75	125	3
…			
145	275	45	2
…			
288	425	195	4
289	–	–	0

**Table 4:** Determined modal loss factors $\eta_{n}$, H- for healthy plate and D for plate with delamination.

Mode	1	2	3	4	5	6	7	Average
H	0.0036	0.0143	0.0546	0.0321	0.0238	0.0226	0.0309	0.026
D	0.0022	0.0027	0.0297	0.0220	0.0016	0.0258	0.0478	0.019

**Table 5:** MFC material properties provided by Smart Materials [36].

Elastic constants	*E*_33_*,* GPa	*E*_11_*,* GPa	*G*_31_*,* GPa	*ν*_31_, –	*ν*_13_, –
	30.34	15.86	5.16	0.31	0.16
Piezoelectric constants	*d*_33_*,* pm/V	*d*_32_*,* pm/V	*ε^T^_11_*, –	*ε^T^_22_*, –	*ε^T^_33_*, –
	467	−210	712	1.7	737

**Table 6:** The numerically determined resonant frequencies (Hz) and corresponding modes.

No.	Mode	Healthy	Damaged	Δ
1	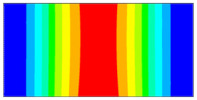	64.5	64.25	−0.4
2	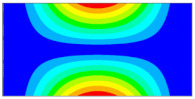	70	70	0.0
3	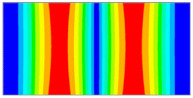	179	178	−0.6
4	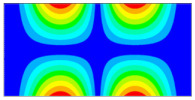	183.75	184.25	0.3
5	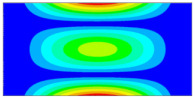	225.25	224	−0.6
6	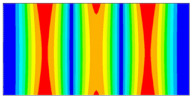	297	295.75	−0.4
7	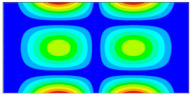	352	354	0.6

**Table 7:** The results of classification for experimental data for the k-NN classifier trained using FEM data plus the addition of three sets of experimental data according to the first learning scheme.

Mode 1		Mode 2		Mode 3		Mode 4		Mode 5		Mode 6		Mode 7		Sum	
18	0	9	1	18	9	18	0	18	0	18	0	18	0	117	10
0	18	9	17	0	9	0	18	0	18	0	18	0	18	9	116

**Table 8:** Overall classification results for the presented research.

Learning Scheme	Training Data Set	Predictive Performance	Test Data Set
First	FEM data	99.0%	FEM data
Second	FEM data	98.3% (detection and localization 97.3%)	FEM data
First	FEM data and Exp. Data	92.5%	Exp. data
Second	FEM data and Exp. Data	100% (detection and localization 92.5%)	Exp. data
Second—higher precision of localization	FEM data and Exp. Data	98.4% (detection and localization 91%)	Exp. data

**Figure A2:**
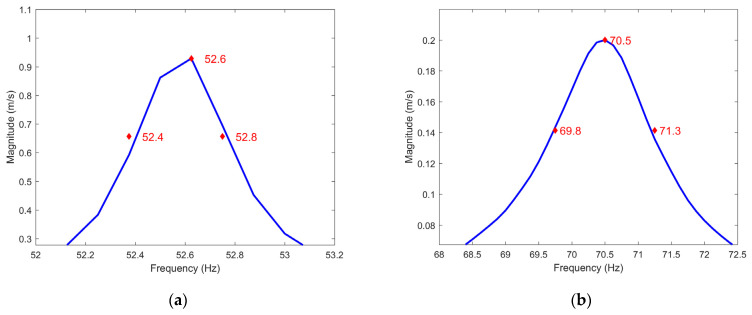
The half-bandwidth method for modal loss factor extraction for (**a**) the first mode and (**b**) the second mode (see Table 2 for details).

**Figure A3:**
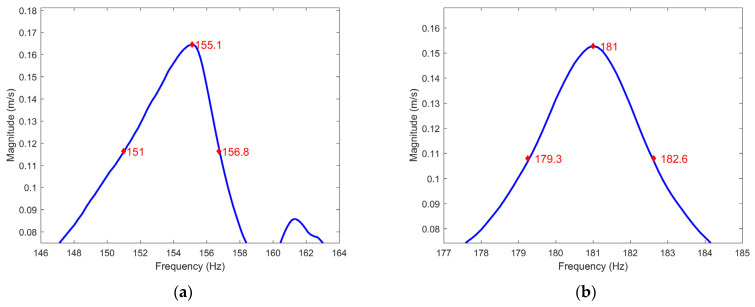
The half-bandwidth method for modal loss factor extraction for (**a**) the third mode and (**b**) the fourth mode (see Table 2 for details).

**Figure A4:**
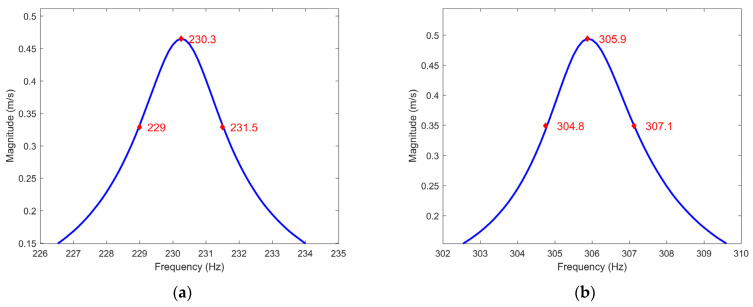
The half-bandwidth method for modal loss factor extraction for (**a**) 5th mode; (**b**) 6th mode (see Table 2 for details).

**Figure A5:**
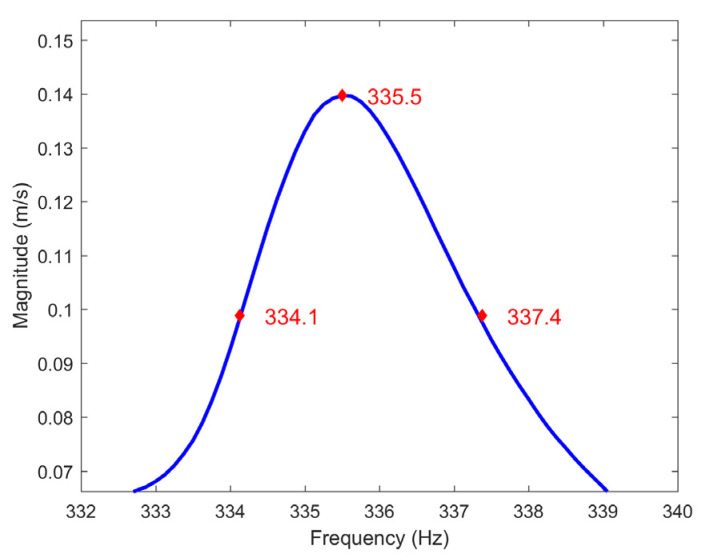
The half-bandwidth method for modal loss factor extraction for the seventh mode (see Table 2 for details).

## Data Availability

The data that support the findings of this study are available from the first author, Sandris Ručevskis, upon reasonable request.

## Appendix A

Tables of strain sensor data for the undamaged plate excited with different amplitudes.

**Table A1:** Strain sensor data for the undamaged plate excited with 1 V amplitude.

		Sensor #							
Mode	Strain	1	2	3	4	5	6	7	8
1	µm/m	10.77	12.91	21.28	16.36	4.39	19.59	14.34	6.67
	scaled	1.00	1.20	1.98	1.52	1.00	4.46	3.26	1.52
2	µm/m	23.41	33.91	46.78	2.73	7.33	22.98	17.11	10.45
	scaled	1.00	1.45	2.00	0.12	1.00	3.14	2.34	1.43
3	µm/m	11.48	33.36	56.01	41.22	20.26	54.07	39.68	24.95
	scaled	1.00	2.91	4.88	3.59	1.00	2.67	1.96	1.23
4	µm/m	13.60	31.88	65.25	42.90	21.52	57.51	42.13	26.61
	scaled	1.00	2.34	4.80	3.15	1.00	2.67	1.96	1.24
5	µm/m	12.09	44.29	76.86	58.26	26.98	73.09	53.64	33.72
	scaled	1.00	3.66	6.35	4.82	1.00	2.71	1.99	1.25
6	µm/m	13.21	50.04	86.12	65.34	30.80	82.46	60.73	38.09
	scaled	1.00	3.79	6.52	4.95	1.00	2.68	1.97	1.24
7	µm/m	12.72	50.09	86.37	64.29	30.35	81.33	59.97	37.62
	scaled	1.00	3.94	6.79	5.05	1.00	2.68	1.98	1.24

**Table A2:** Strain sensor data for the undamaged plate excited with 2 V amplitude.

		Sensor #							
Mode	Strain	1	2	3	4	5	6	7	8
1	µm/m	25.49	28.22	43.87	33.86	14.69	39.79	29.19	18.32
	scaled	1.00	1.11	1.72	1.33	1.00	2.71	1.99	1.25
2	µm/m	55.47	72.81	98.74	18.35	17.25	46.37	34.73	21.68
	scaled	1.00	1.31	1.78	0.33	1.00	2.69	2.01	1.26
3	µm/m	24.15	67.58	112.73	82.31	40.83	108.96	79.90	50.22
	scaled	1.00	2.80	4.67	3.41	1.00	2.67	1.96	1.23
4	µm/m	26.95	64.47	131.13	86.78	43.28	115.88	84.86	53.57
	scaled	1.00	2.39	4.86	3.22	1.00	2.68	1.96	1.24
5	µm/m	24.00	88.74	154.45	116.84	53.94	146.76	107.60	67.58
	scaled	1.00	3.70	6.43	4.87	1.00	2.72	1.99	1.25
6	µm/m	25.69	100.41	171.95	130.24	61.96	165.49	121.75	76.47
	scaled	1.00	3.91	6.69	5.07	1.00	2.67	1.96	1.23
7	µm/m	25.27	100.79	173.41	128.91	61.01	163.53	120.65	75.66
	scaled	1.00	3.99	6.86	5.10	1.00	2.68	1.98	1.24

**Table A3:** Strain sensor data for the undamaged plate excited with 3 V amplitude.

		Sensor #							
Mode	Strain	1	2	3	4	5	6	7	8
1	µm/m	41.62	43.36	66.83	51.51	22.16	59.46	43.52	27.45
	scaled	1.00	1.04	1.61	1.24	1.00	2.68	1.96	1.24
2	µm/m	84.82	115.15	155.91	15.62	25.56	69.28	52.02	32.40
	scaled	1.00	1.36	1.84	0.18	1.00	2.71	2.04	1.27
3	µm/m	36.75	101.70	169.33	123.34	61.29	163.63	120.01	75.43
	scaled	1.00	2.77	4.61	3.36	1.00	2.67	1.96	1.23
4	µm/m	39.57	97.27	196.10	130.74	64.89	173.98	127.43	80.42
	scaled	1.00	2.46	4.96	3.30	1.00	2.68	1.96	1.24
5	µm/m	35.97	133.13	232.05	175.37	80.89	220.35	161.52	101.42
	scaled	1.00	3.70	6.45	4.88	1.00	2.72	2.00	1.25
6	µm/m	38.12	151.16	258.02	195.67	93.30	249.37	183.23	115.25
	scaled	1.00	3.97	6.77	5.13	1.00	2.67	1.96	1.24
7	µm/m	38.67	151.17	259.72	194.22	91.63	245.69	181.35	113.83
	scaled	1.00	3.91	6.72	5.02	1.00	2.68	1.98	1.24

## Appendix B

This appendix contains the MAC results comparing strain data obtained between two experimental setups. Both experimental setups, discussed in Section 4.2, include strain data measured on plates excited by peak-to-peak vibration amplitudes of 1, 2 and 3 V; hence, a total of six mode shapes are compared. Cases 1 to 3 correspond to the first experimental setup with excitation amplitudes of 1, 2 and 3 V, respectively, while Cases 4 to 6 correspond to the second experimental setup with the according vibration amplitudes. Similar to the MAC plots in Figure 14, the mode shapes of the first two resonant frequencies are the least consistent, while the mode shapes of the higher resonant frequencies indicate very good agreement between both experimental setups.

## Appendix D

**Table A4:** Hyperparameter optimization results for the k-NN classifier trained for the first mode of the second learning scheme (a higher precision of localization).

Iteration	Evaluation Result	Objective	Objective Runtime	Best So Far (Observed)	Best So Far (Estimated)	Number of Neighbors	Distance
1	Best	0.2865	0.1525	0.286	0.2865	62	Cosine
2	Accept	0.8889	0.1097	0.286	0.3104	3	Hamming
3	Accept	0.7129	0.3976	0.286	0.3074	846	Chebyshev
4	Error	NaN	0.0681	NaN	0.3074	140	Mahalanobis
5	Best	0.0094	0.0863	0.009	0.0348	11	Minkowski
6	Accept	0.3260	0.1101	0.009	0.0456	80	Minkowski
7	Best	0	0.0841	0	0.0000	4	Minkowski
8	Accept	0	0.1226	0	0.0000	1	Cosine
9	Accept	0	0.1006	0	−0.0001	3	Cosine
10	Error	NaN	0.1196	0	−0.0001	1	Euclidean
11	Accept	0	0.1027	0	−0.0001	1	Chebyshev
12	Accept	0	0.0770	0	−0.0001	4	Chebyshev
13	Accept	0.0031	0.0963	0	−0.0001	1	Correlation
14	Accept	0.1865	0.1235	0	−0.0001	17	Correlation
15	Accept	0.8056	0.5147	0	−0.0001	1437	Correlation
16	Accept	0	0.0854	0	−0.0001	1	City block
17	Accept	0.1295	0.1102	0	−0.0001	17	City block
18	Accept	0.7604	0.4976	0	−0.0001	1424	City block
19	Accept	0	0.0829	0	−0.0001	3	City block
20	Accept	0	0.0851	0	−0.0001	1	Euclidean
21	Accept	0.1611	0.1017	0	−0.0001	22	Euclidean
22	Accept	0.6080	0.3131	0	−0.0001	1	Spearman
23	Accept	0.8611	0.4804	0	−0.0001	1434	Jaccard
24	Accept	0.7722	0.5063	0	−0.0001	1431	Euclidean
25	Accept	0	0.0835	0	−0.0002	3	Euclidean
26	Accept	0.8191	0.7128	0	−0.0002	1436	Spearman
27	Accept	0.8889	0.1358	0	−0.0002	1	Jaccard
28	Accept	0	0.0858	0	−0.0009	2	Chebyshev
29	Accept	0.0024	0.1024	0	−0.0010	2	Correlation
30	Accept	0.8611	0.4860	0	−0.0010	1439	Hamming
